# The relationship between trust in primary healthcare providers among patients with diabetes and levels of depression and anxiety

**DOI:** 10.1371/journal.pone.0239035

**Published:** 2020-09-11

**Authors:** Yazed AlRuthia, Monira Alwhaibi, Haya Almalag, Latifa Almosabhi, Majed Almuhaya, Ibrahim Sales, Ahmed Abdulrahman Albassam, Fawaz Abdullah Alharbi, Wael Mansy, Adel S. Bashatah, Yousif Asiri

**Affiliations:** 1 Department of Clinical Pharmacy, College of Pharmacy, King Saud University, Riyadh, Saudi Arabia; 2 Pharmacoeconomics Research Unit, College of Pharmacy, King Saud University, Riyadh, Saudi Arabia; 3 Medication Safety Research Chair, College of Pharmacy, King Saud University, Riyadh, Saudi Arabia; 4 Department of Clinical Pharmacy, College of Pharmacy, Prince Sattam Bin Abdulaziz University, Alkharj, Saudi Arabia; 5 Drug Information and Poison Centre, Alansar Hospital, Medina, Saudi Arabia; 6 Department of Nursing Education and Administration, College of Nursing, King Saud University, Riyadh, Saudi Arabia; University of Wisconsin Madison School of Pharmacy, UNITED STATES

## Abstract

**Background:**

Trust in healthcare providers has been shown to improve several clinical and patient-reported outcomes. However, its relationship with depression and anxiety has not been investigated among patients with chronic health conditions, such as diabetes. Therefore, the aim of this study was to examine whether trust in primary care physicians among patients with diabetes is associated with their levels of depression and/or anxiety.

**Method:**

Adult patients (≥18 years) with a diagnosis of diabetes, confirmed through their electronic health records, were recruited and interviewed from the primary care clinics of three public hospitals. Patient trust in primary care physicians was assessed using the Health Care Relationship (HCR) Trust scale. Depression and anxiety were assessed using the Patient Health Questionnaire 9-item (PHQ-9) and Generalized Anxiety Disorder 7-item (GAD-7), respectively. Two multiple linear regression analyses were conducted to examine the association of HCR-Trust scores with PHQ-9 and GAD-7 scores controlling for age, sex, Charlson comorbidity index score, education, health literacy, annual income, nationality, duration of illness, and research site.

**Results:**

The number of patients who agreed to be interviewed was 367. Most of the participants were female (61.54%) and Saudi (92.86%). High HCR-Trust scores were negatively associated with PHQ-9 scores (β = -0.18; 95% CI: -0.23 –-0.13; *P* = < .0001), and GAD-7 scores (β = -0.17; 95% CI: -0.22– -0.12; *P* = < .0001).

**Conclusions:**

Trust in primary care physicians among patients with diabetes was associated with lower levels of depression and anxiety. Therefore, healthcare providers should adopt a patient-centered care approach that fosters trust in the relationship between their patients and themselves. Further research should explore whether interventions that foster shared decision-making and trust in healthcare providers might also improve the health outcomes of patients with diabetes.

## Introduction

Depression and anxiety are common mental disorders that are prevalent among different patient populations [1], and patients with chronic health conditions, such as diabetes, have a greater risk of depression and/or anxiety compared to their healthy counterparts [2, 3]. The higher prevalence of depression and/or anxiety among patients with chronic illnesses can be attributable to a multitude of factors, but most notably, the high burden of illness [4, 5]. This is quite evident among patients with diabetes, as they are likely to suffer from serious microvascular (diabetic nephropathy, neuropathy, and retinopathy) and macrovascular (coronary artery disease, peripheral arterial disease, and stroke) complications that worsen their disease burden and increase their risk of having both depression and anxiety [6]. Depression and anxiety are associated with lower adherence to life-saving anti-hyperglycemic medications among patients with diabetes [7, 8]. Depression and/or anxiety also have a negative impact on patient health-related quality of life (HRQoL) and healthcare utilization and costs [9–11]. This is a concern given the rising prevalence rate of diabetes, which was estimated to be 8.4% among the adult population (≥18 years) globally in 2017, compared to 4.7% in 1980 [12, 13]. In Saudi Arabia, the prevalence of diabetes is approximately 20%, which is one of the highest rates in the world, and the incidence rate is increasing by an average of 9% annually [14, 15]. Therefore, screening patients with diabetes for depression and anxiety is important to ensure effective management of diabetes.

The immense personal and economic burden of depression and anxiety on patients with diabetes necessitates involving them in the healthcare decision-making process to achieve patient autonomy [16]. However, in order to achieve patient autonomy, patients need to have a strong trust in their healthcare providers [17]. Patient trust in healthcare providers has a positive correlation with their adherence to prescription medications and improved self-care [18, 19], and medication knowledge among patients with diabetes [20]. Moreover, trust in healthcare providers is positively associated with glycemic control and the physical domain of HRQoL for patients with diabetes [21].

Although depressed or anxious patients tend to be less satisfied with their primary care services, trust in healthcare providers, as an attribute of patient satisfaction with healthcare providers, has not been adequately investigated [22]. Moreover, though small to moderate correlations have been found in studies that examined the relationship between patient trust in healthcare providers and different health outcomes, there is evidence of an upward bias in many of these studies [23]. Robinson et al. has developed a middle-range theory to explain the evolution of trust in the ongoing healthcare provider-patient relationship in the context of chronic health conditions, such as diabetes, using the theoretical coalescence method [24]. Thus, the findings of disparate studies that examined the relationship between trust in healthcare providers and different health outcomes, particularly mental health outcomes, in the context of chronic illness were systematically integrated resulting in a complex middle-range theory. Based on this newly developed theory, there are three distinct stages in the relationship between healthcare providers and patients in which three types of trust are involved (e.g., naïve trust, distrust, and informed trust). Moreover, four aspects of the healthcare providers’ behaviors that foster informed trust, which is associated with improved health outcomes, have been identified. First, “curious listening” which reconciles attentive listening with asking questions that encourage patients to reflect their concerns. Second, “compassionate stranger” which entails being close enough to be touched by the patient’s experience but objective at the same time. Third, being “nonjudgmental collaborator” which entails providing patients with constructive feedback and avoiding any negative or destructive comments. Fourth, encouraging patients to focus on their strengths and adopting healthy lifestyles to cope with their illness rather than focusing on the disease only [24]. By adopting these behavioral attributes an informed trust is formed and solidified between patients and their healthcare providers. This informed trust improves the patients’ self-confidence in managing their own health conditions, such as diabetes, and may lead to an improvement in their mental health [24]. Therefore, this theory provides a better framework to understand how patients’ trust in their healthcare providers can play a pivotal role in improving different health outcomes including the mental well-being of patients with chronic health conditions, such as diabetes [24]. Despite the fact that patients with diabetes have a high likelihood of depression and anxiety and a low likelihood of receiving care for their depressive and anxiety symptoms [25], no study so far has examined the relationship between trust in healthcare providers and depression and/or anxiety in this population [23]. Therefore, this study aimed to examine the relationship between levels of depression and anxiety of patients with diabetes and trust in their primary healthcare providers.

## Materials and methods

### Study design and data source

This was a multi-center cross-sectional study that was conducted in the primary care clinics of three public hospitals in Saudi Arabia. The Saudi government’s healthcare system provides free healthcare to all citizens as well as legal residents and their dependents who are employed in the government sector. However, citizens and legal residents who are working in the private sector are not eligible to receive care from public hospitals, as they should be provided health insurance coverage through their employers [26].

Study participants’ level of depression was assessed using the validated Arabic version of the Patient Health Questionnaire 9-item (PHQ-9) [26]. The PHQ-9 consists of nine items that are rated from 0 to 3, yielding a total possible score of 27, with higher scores indicating more severe depression [27]. The participants’ level of anxiety was assessed using the Arabic version of the Generalized Anxiety Disorder 7-item (GAD-7) [28]. The GAD-7 consists of seven items that are rated from 0 to 3, yielding a total possible score of 21, with higher scores indicating more severe anxiety [28]. Trust in healthcare providers was assessed using the validated Arabic version of the Health Care Relationship (HCR) Trust scale, which consists of 13 statements related to patients’ trust in their healthcare providers [20, 29]. Each statement is rated on a scale from 0 to 4, yielding a total possible score of 52, with higher scores indicating greater trust in healthcare providers [20, 29]. In order to protect patient confidentiality, no personal identifiers were collected. The study adhered to the Declaration of Helsinki’s ethical principles for medical research [30]. Approval of the study was received from the Institutional Review Boards of the College of Medicine at King Saud University, Riyadh, Saudi Arabia, the College of Medicine at Prince Sattam bin Abdulaziz University, Al-Kharj, Saudi Arabia, and Alansaar Hospital, Almadina, Saudi Arabia.

### Study population

Patients were recruited from the primary care clinics of three public hospitals in Saudi Arabia between May 2016 and November 2018. Adult patients (≥18 years) whose diabetes diagnosis was documented in their electronic healthcare records, and did not have any malignancies were eligible for participation. Patients without electronic health records and those with mental illness (e.g., schizophrenia, bipolar disorder, major depressive disorder) or taking any psychotropic medications, as verified by reviewing patients’ electronic health records, were excluded from participation. The medical characteristics (e.g., medical conditions, number of prescription medications, and duration of illness) of patients were collected from their electronic health records. A list of eligible patients was created and patient interviews were scheduled on their appointment days with their primary care physicians. While waiting for their appointment, patients were approached by pharmacy interns who were trained to conduct patient interviews by their clinical preceptors. The patients were asked whether they would be interested in participating in a research project that would take around 15–20 minutes after seeing their primary care physician. Those who agreed to participate were taken to a private meeting room and presented with a written consent form that explained the purpose of the study and patients’ right to withdraw from the study at any time during the interview. After signing the consent form, the interviews started by collecting patients’ sociodemographic characteristics (e.g., age, sex, annual income, nationality, education, and health literacy). Patients were screened for health literacy using the Arabic version of the single item literacy screener (SILS), which consists of one question inquiring about the patient’s ability to understand the instructions in a prescription drug leaflet without help from anyone [31, 32]. Patients who indicated that they never or rarely need help from someone were considered to have good or adequate health literacy, and those who indicated that they need help sometimes, often, or always were considered to have limited or marginal health literacy [31, 32]. In order to assess comorbidities, the Charlson Comorbidity Index (CCI) was calculated. The CCI assigns different scores to 17 health conditions based on the disease severity (e.g., cancer vs. diabetes) [33].

### Statistical analysis

Descriptive statistics, including frequencies, percentages, and means were used to describe the baseline characteristics of the participants. Two multiple linear regression models were conducted to examine whether participants’ trust in health care providers (HCR-Trust score) was associated with their depression (PHQ-9 score) or anxiety (GAD-7 score) levels. Determinants of depression, including age, sex, CCI score, health literacy, education, annual income, duration of illness, and nationality as a proxy for ethnicity, were controlled for in both regression analyses [34]. The minimum sample size was estimated to be 359 patients using Cohen’s statistical power analysis for multiple linear regression, based on α = 0.05, β = 0.2 (power = 80%), effect size (f^2^ = 0.02), and nine predictor variables [35]. Statistical significance was established at α = 0.05. All statistical analyses were conducted using SAS statistical software (version 9.2, SAS Institute Inc., Cary, NC, USA).

## Results

Out of 475 patients who were approached to participate in the study, 364 agreed to participate and were included in the analysis. The majority of the patients (94.23%) had type 2 diabetes. The mean age of the participants was almost 54 years. Approximately two-thirds of the participants were female (61.54%), and the majority were Saudi (92.86%). Their mean CCI score was 2.26, and their mean duration of illness (i.e., diabetes) was approximately 12 years. The participants’ mean number of prescription medications was 5. About 20% of the participants did not have any formal education and were unable to read or write; however, almost 47% of them had a high school degree or above. Almost 52% of the participants had limited health literacy. Most of the participants (59%) had an annual income of more than $19,200, and about 41% had an annual income of $19,200 or less. The patients’ mean HCR-Trust score was almost 37, and their mean PHQ-9 and GAD-7 scores were approximately 8 and 6, respectively (Table 1).

**Table 1:** Baseline characteristics of the patients with diabetes.

Characteristic	N = 364
**Sex, n (%)**	
Male	140 (38.46)
Female	224 (61.54)
**Nationality, n (%)**	
Saudi	338 (92.86)
Non-Saudi	26 (7.14)
**Education, n (%)**	
No formal education (unable to read or write)	73 (20.05)
Elementary school (1–6 years)	65 (17.86)
Intermediate school (7–9 years)	56 (15.38)
High school (10–12 years)	63(17.31)
Some college or college degree (13–16 years)	96 (26.37)
Postgraduate degree (≥17 years)	11 (3.02)
**Health literacy, n (%)**	
Marginal/limited	188 (51.65)
Good	176 (48.35)
**Annual income, n (%)**	
< $9600	87 (23.9)
$9600-≤$19,200	62 (17.03)
>$19,200-≤$31,992	99 (27.2)
>$31,992-≤$48,000	63 (17.31)
>$48,000-≤$63,996	36 (9.89)
>$63,996-≤$79,992	9 (2.47)
> $79,992	8 (2.2)
**Age, Mean ± SD**	53.66 ± 12
**Health Care Relationship Trust Scale (HCRT), Mean ± SD**	36.56 ± 11.52
**Patient Health Questionnaire-9 (PHQ-9), Mean ± SD**	8.34 ± 6.45
**Generalized Anxiety Disorder-7 (GAD-7), Mean ± SD**	5.81 ± 5.62
**Number of medications**	5.17 ± 4.25
**Charlson Comorbidity Index score, Mean ± SD**	2.26 ± 1.15
**Illness duration, Mean ± SD**	11.97 ± 6.7

N: Number; SD: Standard Deviation

Table 2 shows the association between PHQ-9 and HCR-Trust. Patients with high HCR-Trust scores were less likely to have high PHQ-9 scores (β = -0.18, 95% CI = -0.23 –-0.13, *P* < .0001), controlling for age, sex, CCI score, health literacy, education, annual income, illness duration, nationality as a proxy for ethnicity, and research site. Female patients were more likely to have a higher PHQ-9 score than their male counterparts (β = 3.21, 95% CI = 1.93–4.49, *P* < .0001). Annual income was negatively associated with PHQ-9 score (β = -0.60, 95% CI = -1.06 –-0.13, *P* = 0.01). Table 3 shows the association between GAD-7 and HCR-Trust. Patients with high HCR-Trust scores were less likely to have high GAD-7 scores (β = -0.17, 95% CI = -0.22– -0.12, *P* < .0001), controlling for age, sex, CCI score, health literacy, education, annual income, illness duration, nationality, and research site. Female patients were more likely to have a higher GAD-7 score than their male counterparts (β = 1.97, 95% CI = 0.83–3.11, *P* = 0.001). On the other hand, annual income was negatively associated with GAD-7 scores (β = -0.86, 95% CI = -1.25 –-0.40, *P* = < .0001).

**Table 2:** Multiple linear regression for the association between patient health questionnaire-9 (PHQ-9) scores and health care relationship (HCR) Trust scores.

Variable	β estimate	P-value	95% Confidence interval	
HCRT	-0.18	< .0001*	-0.23	-0.13
Age	-0.03	0.375	-0.1	0.04
Charlson Comorbidity Index score	0.38	0.287	-0.32	1.10
Health literacy	0.08	0.89	-1.20	1.36
Sex	3.21	< .0001*	1.93	4.49
Education	-0.43	0.103	-0.94	0.08
Annual income	-0.60	0.011*	-1.06	-0.13
Illness duration	0.06	0.133	-0.02	0.15
Nationality	-0.24	0.829	-2.52	2.02

*P<0.05

**Table 3:** Multiple linear regression for the association between generalized anxiety disorder-7 (GAD-7) scores and health care relationship (HCR) trust scores.

Variable	β estimate	P-value	95% Confidence interval	
HCRT	-0.17	< .0001*	-0.22	-0.12
Age	-0.02	0.489	-0.08	0.04
Charlson Comorbidity Index score	-0.18	0.575	-0.82	0.45
Health literacy	0.14	0.80	-0.99	1.29
Sex	1.97	0.001*	0.83	3.11
Education	-0.08	0.726	-0.544	0.37
Annual income	-0.86	< .0001*	-1.27	-0.45
Illness duration	0.03	0.447	-0.048	0.109
Nationality	0.55	0.593	-1.47	2.58

*P<0.05

## Discussion

The relationship between chronic illness, such as diabetes, and depression has been established in numerous studies [4–6]. Trust in healthcare providers among patients with diabetes is associated with improved adherence level, self-care, physical HRQoL, and medication knowledge [18–21]. Moreover, trust in healthcare providers is positively associated with a variety of objective and observer-rated health outcomes [23]. However, the Robinson’s middle-range theory we used to describe the relationship between trust in healthcare providers and chronic illness with regard to trust’s impact on depression and/or anxiety among patients with diabetes was not previously operationalized [23, 24]. Therefore, the findings of this study suggest a positive relationship between trust in healthcare providers and mental health (i.e., depression and anxiety) among patients with diabetes. Moreover, they highlight the importance of practicing patient-centered care, in which trust is a fundamental element.

Healthcare providers who engage in building a trusting relationship with their patients are more likely to have patients with better mental health. Although multiple studies have examined the relationship between patient trust in healthcare providers and different health outcomes among patients with chronic diseases, such as diabetes, no study to date has examined the relationship between patient trust in healthcare providers and their levels of depression and/or anxiety [18–21]. This potential relationship between patients’ trust in healthcare providers and their levels of depression and anxiety in the current study remained significant even after controlling for several covariates, including age, CCI score, health literacy, sex, education, annual income, illness duration, research site, and nationality. This is consistent with the results of the few studies that have explored the relationship between patient trust in healthcare providers and different clinical and patient-reported outcomes [23]. However, the glycemic control (e.g., HbA1C) which is one of the important confounders that is believed to have an impact on patient level of depression and/or anxiety was not controlled for.

Arguably, trust is an integral part of patient satisfaction with healthcare providers and the health system, overall. Lee King and colleagues, who investigated the effects of positive patient-provider relationships and perceived clinical outcomes in the treatment of depression [36], found patients’ trust in their healthcare providers was significantly associated with the core elements of a cordial patient-provider relationship. A previously published study of patients with diabetes found those who had lower levels of depression and/or anxiety were more likely to be satisfied with their primary care services [22]. However, what makes the findings of the current study interesting is that it directly explored the relationship of patients’ trust in their healthcare providers with depression and anxiety levels instead of examining it as an embedded attribute of patient satisfaction with healthcare providers. In addition, regular screening of patients with diabetes for depression and anxiety, and attentive listening to their concerns, which is one of the important attributes of patient-centered care, may improve patients’ trust in their healthcare providers and eventually lead to better health outcomes [25]. This cannot be emphasized enough given the negative impact of depression and anxiety on a multitude of health outcomes, including medication adherence, which is crucial for preventing serious complications of diabetes, such as myocardial infarction and stroke [7, 8].

Showing respect for patients’ cultural values and social norms has also been linked to higher levels of patient trust in their healthcare providers [23]. This is very important in a conservative culture, such as Saudi Arabia, where women, for example, are expected to be highly respected for their modesty and listened to by their healthcare providers more often than men are. Unfortunately, female patients with diabetes were more likely to have higher levels of both depression and anxiety, which is consistent with the preponderance of the evidence that suggests higher levels of depression and anxiety among women with diabetes [37]. Moreover, patients with higher annual incomes were less likely to have high depression and anxiety levels, which is also consistent with previously published studies [34]. The duration of illness or diabetes is believed to be associated with a higher likelihood of having higher levels of depression and anxiety [38], however, this relationship was not found to be significant among the study sample. These findings suggest that determinants of depression and/or anxiety, such as gender and income, should be controlled for in any analysis of the relationship between depression and/or anxiety and any chronic illnesses. In addition, the findings of this study highlight a potential role of trust in the management of depression among patients with diabetes. The effective management of depression among patients with diabetes does not only improve patients’ trust in their primary health care providers, but it also enhances their self-efficacy and glycemic control [39]. Therefore, building a trusting working relationship between patients and their healthcare providers is essential for managing depression either through pharmacologic (e.g., antidepressants) or non-pharmacologic (e.g., cognitive behavioral therapy) approaches. The application of cognitive behavioral therapy (CBT) in the management of depression, which has shown promising results in improving depression among patients with diabetes, entails a high level of trust between patients and their healthcare providers since it involves the identification of patients’ false beliefs, which negatively influence their mental well-being [39].

Although this study examined the relationship between patients with diabetes levels of depression and anxiety and their levels of trust in their healthcare providers using validated scales, several limitations should be acknowledged. First, this was a cross-sectional study, so causality cannot be established between patient trust in healthcare providers and their levels of depression and anxiety [40]. Additionally, despite the fact that the study was conducted in three health centers, it used convenience sampling, which limits the generalizability of the findings [41]. Moreover, the possibility of recall bias cannot be ruled out since patients were asked to recall depression and/or anxiety symptoms within the past two weeks [42]. Also, some factors that are associated with depression and/or anxiety, such as the severity of illness and marital status, were not controlled for [34]. In addition, the ethnicity of the participants, which is believed to be one of the determinants of mental health [34], was controlled for using a proxy variable (e.g., nationality) due to the difficulty in obtaining such information from the participants in a reserved culture. Furthermore, the questionnaires (HCR-Trust scale, PHQ-9, and GAD-7) were not self-administered, but were part of a patient interview. This mode of questionnaire administration, although more convenient for the patient, increases the risk of yes-saying bias, interviewer bias, and social desirability bias [43]. Additionally, about 23% of the patients who were invited refused to participate; those patients may have different medical and sociodemographic characteristics that may change the findings of this study if they were included in the analysis. Finally, glycemic control as measured by glycated hemoglobin (HbA1c), which can influence the relationship between trust in healthcare providers and depression and/or anxiety levels among patients with diabetes, was not controlled for [21]. However, this was mainly due to the incompleteness of information in the patients’ electronic health records.

In conclusion, the findings of this study should encourage healthcare providers to build a trusting relationship with their patients, particularly those with chronic health conditions, such as diabetes. This entails the adoption of patient-centered practice where patients should be empowered by attentively listening to their concerns, explaining their health conditions and treatment options to them in lay terms, and engaging them afterwards in the health decision-making process to earn their trust. Gaining the trust of patients with diabetes is very important because it may improve their mental well-being, as this study suggests, and improvement in depression and anxiety symptoms in patients with diabetes is linked to positive health outcomes. Finally, future studies should examine the relationship between patient trust in healthcare providers using more robust study designs with different patient populations.

## Supporting information

S1 File De-identified data. (XLSX)
